# Implementing exercise recommendations into clinical practice—new findings from mental health professionals' and patients' perspectives in a university psychiatric setting

**DOI:** 10.3389/fspor.2024.1336356

**Published:** 2024-05-20

**Authors:** Anna Hirschbeck, David Kossmann, Hannah Schwegler, Sophie-Kathrin Greiner, Alkomiet Hasan, Astrid Roeh

**Affiliations:** Department of Psychiatry, Psychotherapy and Psychosomatics, Medical Faculty, Bezirkskrankenhaus Augsburg, University of Augsburg, Augsburg, Germany

**Keywords:** barriers, exercise recommendations, knowledge, mental illnesses, mental health professional, physical activity behavior

## Abstract

**Introduction:**

To date, concrete recommendations for physical activity in psychiatric treatments are limited. Thus, we evaluated knowledge, barriers and beliefs associated with exercise prescription of mental health professionals (MHP) to people with mental illnesses. We aimed to identify patients' barriers to exercise participation and to work out options addressing these barriers.

**Methods:**

In our cross-sectional and questionnaire-based investigation, we recruited medical, nursing and therapeutic staff and patients of a psychiatric clinic by email and personal contact. Questionnaires contained the German versions of The Exercise in Mental Illness Questionnaire (EMIQ-G) and the International Physical Activity Questionnaires (IPAQ).

**Results:**

We included 100 MHP and 100 patients. MHP had significantly more knowledge regarding positive effects of exercise on general health than patients. Exercise was prescribed mostly (48.4%) or always (37.9%) by MHP. The data showed missing education in exercise prescriptions and different recommendation behavior. Male patients seemed to experience exercise more often as a positive distraction and had lower physical health barriers than females.

**Discussion:**

Physical activity needs to be more integrated in psychiatric treatments. Some strategies as educating MHP and patients regarding potential benefits of exercise via psychoeducative brochures and adapting recommendations to individual symptoms could improve exercise behavior in psychiatric patients.

## 1 Introduction

Regular physical activity is associated with many benefits for mental health (1): a meta-analysis of 25 randomized controlled trials (9 studies with major depressive disorder) was able to show a significant positive effect of physical activity on depressive symptoms (2). Further studies confirmed that aerobic endurance exercise (3) and physical activity in general (4) improve cognitive functioning in people with schizophrenia. Also, anxiety symptoms (5–7) and substance use disorders (8) can beneficially be influenced by physical activity.

Otherwise, data from 168 countries (representing 96.0% of the global population) showed that 27.5% of all adults were not sufficiently physical active (9). Several studies confirmed that sedentary lifestyle may increase the risk of obesity, cardiovascular diseases, diabetes, reduce quality of life and can contribute to the development of mental illnesses (10). The current WHO (World Health Organization) recommendations for physical activity levels in healthy adults (18–65 years) include a weekly amount of at least 150 min of moderate-intense physical activity, 75 min of vigorous-intense physical activity or a combination of moderate- and vigorous-intense exercise. In addition, 2–3 strength units per week of all large muscle groups are recommended (11).

In recent years, there has been increasing interest from researchers and mental health professionals (MHP) in physical activity as a means of improving and preventing mental symptoms (12). However, most patients with mental disorders have lower levels of physical activity compared to a healthy population (13). Individuals with mental disorders experience a range of barriers preventing them from engaging in regular physical activity, such as low mood, high levels of perceived stress, lack of social support and negative beliefs about capabilities (14–16). Therefore, there is a need for feasible and efficacious treatment options to overcome these barriers and to sustainably increase physical activity in patients with mental disorders. For such lifestyle changes towards more physical activity, patients with mental illnesses need the support and advice of MHP (17) because especially physicians, psychologists and nurses play an important role in providing information on health behavior to their patients (18).

To date, concrete recommendations or prescriptions for physical activity in psychological or psychiatric treatments are limited (19). In the US, less than one in three adults with symptoms of depression received advice from a healthcare provider regarding physical activity or exercise (19). This is in contrast to other medical specializations such as cardiology, where physical activity has already been well integrated into clinical therapy with specific recommendations for cardiovascular diseases (20). Physical exercise in mental illnesses can improve not only mental but also physical symptoms at the same time.

The vast majority of MHP in different parts of the world have stated that they hold a positive attitude towards exercise and prescribe it to their patients with mental illnesses (21–25). For example, a German study reported that most MHP recommend physical activity to some (*N *= 82, 54.5%) or all of their patients (*N* = 51, 33.8%) (25) while in an Australian study, Stanton et al. found that 72% of nurses reported prescribing exercise to patients with mental illnesses (24). Several barriers to the implementation of approaches to promote physical activity into clinical standard care in the psychiatric setting have been identified. Some of the main barriers include a lack of knowledge on the benefits of physical activity (26, 27) and lack of formal education or practice in the promotion of physical activity (23, 27, 28). However, most studies did not examine patients with mental disorders themselves, but only health professionals. Therefore, quantitative studies investigating psychiatric patients' perspectives are lacking.

The promotion of physical activity is required to improve daily life functions and to delay the progression of several mental and physical diseases. To take full advantage of the positive effects of physical activity, it is imperative that especially MHP are well placed to identify individual needs for exercise promotion and to support people with mental illness in being physically active.

### 1.1 Aim

In that regard, the aim of the present study was to evaluate knowledge, barriers and beliefs, including the importance, benefits and correlations associated with exercise prescription and recommendation by MHP to people with mental illnesses. Further, we aimed to identify patients' barriers to participation in exercise and to work out options addressing barriers of both MHP and patients. Finally, we aimed to identify sex differences in patients in order to be able to make sex-related conclusions and to give more specific exercise recommendations in clinical treating.

## 2 Methods

This study was a cross-sectional and questionnaire-based investigation into the knowledge, attitudes, beliefs and behaviors of health professionals working with patients with mental illnesses, regarding the role of exercise in the treatment of mental illnesses. In addition, we examined psychiatric patients' knowledge, barriers and expectations regarding the role of physical activity in their daily life and compared both perspectives.

### 2.1 Subjects

MHP of the psychiatric clinic of Psychiatry, Psychotherapy and Psychosomatics of the University Augsburg (Augsburg, Germany) and the oncological clinic of the university hospital Augsburg (CCCA—Comprehensive Cancer Center Augsburg) were recruited by email and personal contact. MHP are all clinical employees who are in direct contact with patients and are employed. In CCCA, MHP work with physically ill patients who often have comorbid mental health symptoms. Patients in the psychiatric clinic of Psychiatry, Psychotherapy and Psychosomatics of the University Augsburg (Augsburg, Germany) were recruited by personal contact without restrictions regarding their psychiatric diagnoses. The study size planning was based on a comparison (independent *t*-test) between mental health professionals and patients and their level of physical activity. With an expected effect size of 0.4, an *α* = 0.05 and a statistical power of 1–*ß* = 0.8, 100 participants must be recruited per group. Thus, a total of 200 participants (100 MHP, 100 patients) were recruited. Our inclusion criteria were sufficient German language, age range between 18 and 65 years, voluntary treatment and ability to consent. The participants who had contradiction for physical activity were not included in the study.

### 2.2 Ethics and registration

The study was conducted in accordance with the guiding principles of the Declaration of Helsinki 2008, local laws and regulations. The study protocol was approved by the ethics committee of the Ludwig-Maximilian University Munich (approval number 21-0462). The study was registered at https://www.drks.de (DRKS-ID: DRKS00025970). All participants provided written informed consent before inclusion in the study.

### 2.3 Instruments

#### 2.3.1 EMIQ

Mental health professionals' knowledge about physical activity in people with mental illnesses was evaluated with the German version of The Exercise in Mental Illness Questionnaire (EMIQ-G) (29). The instrument is a self-report questionnaire containing 65 items assigned to 6 domains: (1) Knowledge on physical activity (2) Beliefs/Attitudes concerning physical activity (3) Recommendation/Prescription behavior (4) Barriers concerning physical activity (5) Physical activity behavior and (6) Demographics (29). As there is no validated version for patients, we adapted the questionnaire for this purpose containing 36 items assigned to 4 domains: (1) Knowledge on physical activity (2) Barriers concerning physical activity (3) Incentives concerning exercise participation (4) Expectations concerning regular exercise (see Supplementary Material S1).

#### 2.3.2 Physical activity

We used the International Physical Activity Questionnaires (IPAQ) to evaluate detailed information about daily activity including physical exercise as well as physical activity in daily routines (30). MET (metabolic equivalent of task minutes) (31) per week were calculated as recommended by Craig et al. (30). There is already a German translation of the International Physical Activity Questionnaire—Short Form (IPAQ-SF) (32). Previous studies revealed high correlations of physical activity measured with accelerometers to the questionnaire (33).

### 2.4 Statistical data analysis

All analyses were conducted using SPSS 29.0 (IBM, Armonk, NY, USA), with a significance level of alpha <0.05. Descriptive statistics were used to report respondent participants' characteristics and answers regarding knowledge, beliefs, behaviors and barriers. Demographic and clinical differences between groups of MHP and patients were assessed using Mann–Whitney-*U* Test due to violation of the normal distribution (Shapiro–Wilk Tests, *p* < 0.05). Homogeneity of variances was tested with the Levene's test. Fisher's-Freeman–Halton Test was used to analyse sex contribution (female, male, divers) between the two groups MHP and patients (34). The effect size *r* for Mann–Whitney-*U* Tests was calculated when comparing the sex differences in patients according to Cohen (35). In a separate analysis and in order to present the knowledge (summed score of the six questions) and the barriers (summed score from the 16 questions), we used the mean response from the levels of agreement questions. Differences in respondents' (MHP vs. patients) summed scores were examined using Mann–Whitney-*U* Test based on Stanton et al. (27). All datasets were screened for plausible values. Only items that were filled out correctly and completely were included in the data analysis.

## 3 Results

### 3.1 Participants characteristics

Of the 100 MHP, 11 were medical doctors, 30 were psychologists, 37 were nurses, 10 were specialized therapists, 7 were physiotherapists or sports therapists and 5 medical doctors were of CCCA. Of the 100 patients, 90 were inpatients and 10 were outpatients at the time of inclusion. All patients had a main diagnosis according to ICD-10 (F1:1, F2:4, F3:92, F4:2, F5:1). Almost all patients (*N* = 83/97) reported taking psychotropic drugs, including antidepressants (78/83), antipsychotics (42/83), anxiolytics (23/83) or stimulants (2/83).

Significant differences between both groups regarding sex, age, BMI, education, and IPAQ are presented in Table 1.

**Table 1:** Baseline characteristics of mental health professionals (MHP) and patients (PA).

	*N* (MHP/PA)	MHP		PA		MHP vs. PA		
						*χ* ^2^	* *	*p*
Sex (male/female/diverse)	200 (100/100)	23/77/0		39/58/3		9.389	* *	0.006
		*M*	*SD*	*M*	*SD*	*U*	*Z*	*p*
Age (years)	191 (97/94)	37.81	12.08	32.56	14.40	3,096.0	−3.833	<0.001
BMI (kg/m^2^)	199 (100/99)	23.66	4.85	25.72	6.53	4,008.5	−2.318	0.020
Education (years)	183 (95/88)	17.14	3.94	13.51	3.15	1,766.5	−6.768	<0.001
IPAQ (MET)	200 (100/100)	5,957.49	4,989.47	2,537.19	2,293.91	2,304.0	−6.587	<0.001

Number of participants (*N*) after elimination of invalid questionnaires or non-existing data; Fischer's-Freeman-Halton Test with presentation of *χ*^2^ and *p*; Mann–Whitney-*U* Test with presentation of *U*, *Z*, *p*; MET (metabolic equivalent of task minutes).

### 3.2 Knowledge regarding the benefits of exercise

Of all MHP (*N *= 100), only 97 participants reported whether they had ever received any formal training in exercise prescription, 3 gave no answer. Of these, only 19 participants (19.6%) had received one and 78 (80.4%) had received none. Additional questions were only asked to participants who had received formal training (*N* = 19). All 19 participants (3 doctors, 5 psychologists, 5 nurses, 6 physiotherapists or sports therapists) referred to their professional education when asked to specify the formal training. Ten participants rated their knowledge of exercise prescription for people with mental illness as “Good” (*N* = 8, 42.1%) or “Excellent” (*N* = 2, 10.5%). Fourteen participants rated their confidence to prescribe exercise to people with mental illness as “Good” (*N* = 9, 47.4%) or “Excellent” (*N* = 5, 26.3%).

Comparing MHP and patients, the results of the Mann–Whitney-*U* Tests showed that MHP knew significantly more regarding the beneficial effects of exercise in cholesterol level (*p* = 0.004), blood pressure (*p* < 0.001), depression (*p* < 0.001) and risk of cancer (*p* < 0.001) than patients. However, basic knowledge seems to be missing., e.g., 18.0% of MHP did not know for sure that physical activity is preventive regarding the development of depression. The results of the sum score (mean response from the six question) revealed that MHP had significantly more knowledge overall than the patients (*U* = 2,609.0, *Z* = −4.775, *p* < 0.001). Detailed information about the participant's response is shown in the Supplementary Table S1.

### 3.3 Mental health professionals' perspective

#### 3.3.1 Beliefs regarding exercise

MHP were asked to rank the importance of several treatment strategies in people with mental illness. Only 75 participants fully completed this item. Medication (*N* = 22, 29.7%) was ranked first most frequently, followed by social support (*N* = 17, 23.0%), electroconvulsive therapy (*N* = 7, 9.5%), social skills training (*N* = 6, 8.1%), psychotherapy (*N* = 5, 6.8%), vocational rehabilitation (*N* = 4, 5.4%), family therapy (*N* = 4, 5.4%), hospitalization (*N* = 3, 4.1%) and light therapy (*N* = 3, 4.1%). Exercise (*N* = 3, 4.1%) was least frequently ranked on first position, but mostly on fourth position (*N* = 27, 36.5%).

#### 3.3.2 Behavior of exercise prescription

##### Frequency of exercise prescription

This question (Q16) only applied to MHP (*N* = 95). 36 respondents (37.9%) reported *Always* prescribing exercise, 46 (48.4%) reported prescribing exercise *Most of the time*, 13 (13.7%) reported prescribing exercise *Occasionally* and none reported *Never* prescribing exercise.

##### Behavior in recommending exercise

Table 2 shows MHP behavior in recommending exercise (explaining to patients what to do and how to do it) to people with mental illnesses. *Brochures* were named very rarely (13.5%) as a possible method prescribing exercise to people with a mental illness. *Personal discussion/recommendation* (88.5%) is used the most, followed by *referral to an exercise professional* (43.8%). The practices of MHP regarding the type, the duration and the intensity of physical activity were very different. However, *aerobic exercise* (92.6%) was named the most as a possible type of exercise training. MHP stated that they most often recommend 20 min (30.9%) or 30 min (31.9%) per session and mostly in a *moderate intensity* (44.6%).

**Table 2:** Behavior (Q17–Q22) regarding exercise for patients with mental illnesses of mental health professionals.

Item	Answer options	Number (valid %)
Q17 Do you undertake a formal assessment of the clients’ suitability for exercise prior to prescribing a program? *N* = 93	Yes	21 (22.6)
	No	72 (77.4)
Q18 When you prescribe exercise to people with a mental illness, what methods do you use? Multiple answers possible. *N* = 96	Personal discussion	85 (88.5)
	Brochures or pamphlets	13 (13.5)
	Referral to community-based programs	15 (15.6)
	Referral to an exercise professional	42 (43.8)
	Nothing specific	9 (9.4)
	Other	21 (21.9)
Q19 When you prescribe exercise to people with a mental illness, how often do you recommend they exercise? *N* = 96	Every day	13 (13.5)
	Most days of the week	26 (27.1)
	Once to twice a week	21 (21.9)
	As often as they feel they can	28 (29.2)
	Other	8 (8.3)
Q20 When you prescribe exercise to people with a mental illness, how hard (what intensity) do you recommend they exercise? *N* = 92	Low intensity	7 (7.6)
	Moderate intensity	41 (44.6)
	Vigorous intensity	2 (2.2)
	At a level that makes them feel good	28 (30.4)
	I do not suggest an intensity	12 (13.0)
	Other	2 (2.2)
Q21 When you prescribe exercise to people with a mental illness, how long do you suggest people try to exercise for at any one time? *N* = 94	10 min per session	7 (7.4)
	20 min per session	29 (30.9)
	30 min per session	30 (31.9)
	60 min per session	4 (4.3)
	As long as they can	9 (9.6)
	Other	15 (16.0)
Q22 When you prescribe exercise to people with a mental illness, what type of exercise do you suggest? Multiple answers possible. *N* = 95	Aerobic exercise	88 (92.6)
	Weight or resistance training	44 (46.3)
	Swimming	64 (67.4)
	Team sports	47 (49.5)
	Combat sports	26 (27.4)
	Relaxation activities (Thai Chi, Yoga)	77 (81.1)
	Other	19 (20.0)

The differences to *N* = 100 were caused by missing values.

#### 3.3.3 Barriers to exercise prescription

MHP responses to statements regarding the barriers to exercise prescription for people with mental illness are shown in Table 3. The data showed low organizational barriers (e.g., Q30 too excessive workload: 50.0% *strongly disagree*; Q31 not part of their job: 52.1% *strongly disagree*).

**Table 3:** Levels of agreement by mental health professionals with statements regarding barriers to exercise prescriptions (Q23–Q36) for patients with mental illnesses.

Item	Absolute numbers (valid %)				
	Atrongly disagree	Disagree	Neither disagree nor agree	Agree	Strongly agree
Q23 Their mental health makes it impossible for them to participate in exercise. *N* = 94	22 (23.4)	48 (51.1)	13 (13.8)	10 (10.6)	1 (1.1)
Q24 I’m concerned exercise might make their condition worse. *N* = 96	52 (54.2)	42 (43.8)	1 (1.0)	1 (1.0)	0
Q25 I’m not interested in prescribing exercise for people with a mental illness. *N* = 96	75 (78.1)	16 (16.7)	3 (3.1)	1 (1.0)	1 (1.0)
Q26 I don’t believe exercise will help people with a mental illness. *N* = 96	79 (82.3)	15 (15.6)	1 (1.0)	1 (1.0)	0
Q27 Their physical health makes it impossible for them to participate in exercise. *N* = 95	29 (30.5)	42 (44.2)	20 (21.1)	4 (4.2)	0
Q28 I’m concerned they might get injured while exercising. *N* = 94	31 (33.0)	43 (45.7)	17 (18.1)	3 (3.2)	0
Q29 People with a mental illness will not adhere to an exercise program. *N* = 94	15 (16.0)	35 (37.2)	35 (37.2)	9 (9.6)	0
Q30 My workload is already too excessive to include prescribing exercise to people with a mental illness. *N* = 94	47 (50.0)	38 (40.4)	5 (5.3)	4 (4.3)	0
Q31 Prescribing exercise to people with a mental illness is not part of my job. *N* = 94	49 (52.1)	32 (34.0)	9 (9.6)	3 (3.2)	1 (1.1)
Q32 I don’t know how to prescribe exercise to people with a mental illness. *N* = 96	39 (40.6)	36 (37.5)	16 (16.7)	5 (5.2)	0
Q33 Prescription of exercise to people with mental illness is best delivered by an exercise professional such as a physiotherapist. *N* = 96	19 (19.8)	37 (38.5)	29 (30.2)	10 (10.4)	1 (1.0)
Q34 I would recommend physical activity to people with mental illness if there was collaboration/support with other medical disciplines. *N* = 96	9 (9.4)	7 (7.3)	19 (19.8)	44 (45.8)	17 (17.7)
Q35 I would recommend physical activity to people with mental illness if physical activity is considered when planning (including prioritizing) patient care. *N* = 96	4 (4.2)	4 (4.2)	28 (29.2)	38 (39.6)	22 (22.9)
Q36 I would recommend physical activity to people with mental illness if I received extensive training in the possibilities of physical activity. *N* = 96	6 (6.3)	10 (10.4)	20 (20.8)	38 (39.6)	22 (22.9)

The differences to *N* = 100 were caused by missing values.

### 3.4 Patients' perspective

For the final analysis and sex comparisons, only patients who indicated male or female were included. The sample size of the sex *diverse* (*N* = 3) was too small for further sex-related comparisons.

#### 3.4.1 Motivating factors and barriers towards exercise

We summarized the questionnaire's categories of incentives and expectations as the motivating factors towards exercise in patients with mental illnesses. In part, we identified significant differences in males and females with a weak to moderate effect. We compared whether the barriers to exercise participation experienced by patients with mental illness corresponded to MHP assumptions regarding these barriers. The statements largely matched, except for the statement that they believe physical activity will not help psychiatric patients. More details can be seen in the Supplementary Table S2.

#### 3.4.2 Sex differences

The results of the Mann–Whitney-*U* Tests revealed that males were more likely to have positive expectations of regular exercise than females [e.g., reduced need in medication (Q26) or positive distraction (Q33)]. Otherwise, female participants expected less improvement in their health values (Q27) than males. Regarding barriers to exercise participation, females showed more negative associations with exercise than males in the statements of well-being (Q12), of confidence (Q18), of safe place (Q21) and of physical health (Q20) [e.g., weight (Q19)]. Social support (Q16) was experienced as a greater barrier by men compared to women. More details can be seen in Table 4.

**Table 4:** Motivating factors (incentives Q23–Q25 and expectations Q26–Q36) and barriers (Q7–Q22) regarding exercise for patients with mental illnesses and comparison between males (m) and females (f) within the patients.

	*N* (m/f)	Male		Female		m vs. f		
		*M*	*SD*	*M*	*SD*	*U*	*Z*	*p*
**Incentive**								
Q23 I would see a financial reward for physical activity (e.g., heal insurance) as an incentive.	92 (37/55)	3.43	1.259	3.71	1.066	901.5	−0.970	0.332
Q24 Detailed guidance on physical activity exercises would make it easier for me to exercise.	92 (37/55)	3.32	1.248	3.53	0.997	934.0	−0.705	0.481
Q25 If I’m regularly supported and accompanied when doing physical activity, it is easier for me to integrate physical activity into my everyday life.	92 (37/55)	3.76	1.234	3.91	0.928	1,000.5	−0.146	0.884
**Expectation**								
Q26 If I’m regularly active in health sports, my need for medication is reduced.	92 (37/55)	3.08	0.894	2.67	0.944	760.0	−2.155	**0** **.** **031**
Q27 If I’m regularly active in health sports, my health values improve.	92 (37/55)	4.32	0.530	3.89	0.737	698.5	−2.877	**0** **.** **004**
Q28 If I’m regularly active in health sports, I meet nice people.	92 (37/55)	3.19	0.739	3.16	1.014	1,002.5	−0.126	0.900
Q29 If I’m regularly active in health sports, my physical well-being improves.	92 (37/55)	4.27	0.732	4.15	0.756	921.5	−0.835	0.404
Q30 If I’m regularly active in health sports, my self-confidence improves.	92 (37/55)	4.03	0.763	3.71	0.896	838.5	−1.568	0.117
Q31 If I’m regularly active in health sports, I can be proud of myself.	91 (36/55)	3.94	0.893	3.91	1.076	959.5	−0.268	0.789
Q32 If I’m regularly active in health sports, I lose weight.	92 (37/55)	3.81	1.050	3.69	0.979	944.5	−0.614	0.539
Q33 Physical activity is a positive distraction for me.	92 (37/55)	4.11	0.737	3.56	1.014	699.0	−2.802	**0** **.** **005**
Q34 My daily routine is better structured through regular physical activity.	92 (37/55)	3.59	0.725	3.35	0.927	853.5	−1.400	0.162
Q35 If I’m regularly active in health sports, I can make a fool of myself.	91 (37/54)	2.24	1.038	2.78	1.298	769.0	−1.915	0.055
Q36 If I’m regularly active in health sports, I can overexert myself.	92 (37/55)	3.08	1.140	3.18	1.156	972.0	−0.384	0.701
**Barrier**								
Q7 My mental health makes it impossible for me to participate in exercise.	92 (37/55)	2.46	1.070	2.89	1.133	794.0	−1.848	0.065
Q8 I’m concerned exercise might make my condition worse.	92 (37/55)	1.76	0.983	1.75	0.985	1,006.5	−0.096	0.923
Q9 I don’t believe exercise will help me with my mental illness.	92 (37/55)	2.24	1.234	2.29	1.197	981.0	−0.304	0.761
Q10 My physical health makes it impossible for me to participate in exercise.	92 (37/55)	1.65	0.824	2.04	1.138	833.0	−1.577	0.115
Q11 I’m concerned I might get injured while exercising.	92 (37/55)	1.92	1.064	2.00	1.186	998.0	−0.166	0.868
Q12 I’m too unwell to exercise.	91 (37/54)	2.38	1.114	2.89	1.110	733.5	−2.218	**0** **.** **027**
Q13 It takes too much time.	92 (37/55)	2.16	1.041	2.60	1.116	791.5	−1.875	0.061
Q14 There is too much stigma attached to having a mental illness.	91 (37/54)	4.00	0.882	4.04	1.009	948.0	−0.435	0.663
Q15 I don’t know what I should do.	91 (37/54)	2.97	1.190	3.19	1.290	907.0	−0.764	0.445
Q16 My friends or family won’t exercise with me.	91 (37/54)	4.05	0.941	3.54	1.161	747.5	−2.136	**0** **.** **033**
Q17 There are too many side effects from the medications.	91 (37/54)	3.24	1.090	3.17	1.178	975.5	−0.197	0.844
Q18 I lack the confidence to do any exercise.	92 (37/55)	2.16	1.214	2.87	1.348	711.5	−2.510	**0** **.** **012**
Q19 I’m too fat to exercise.	92 (37/55)	1.51	0.901	2.15	1.224	666.0	−3.037	**0** **.** **002**
Q20 I have too many physical health problems.	92 (37/55)	1.84	1.093	2.24	0.942	727.5	−2.451	**0** **.** **014**
Q21 There is no safe place for me to exercise.	92 (37/55)	2.00	1.106	2.58	1.301	754.0	−2.179	**0** **.** **029**
Q22 I don’t have any equipment to do exercise with.	92 (37/55)	2.00	1.080	1.98	1.209	972.0	−0.386	0.700
Summed score from the 16 levels of agreement questions of barriers	90 (37/53)	38.35	9.592	42.47	11.281	756.0	−1.839	0.066

The differences to *N* = 100 were caused by missing values.

The significant values in the table are printed in bold.

## 4 Discussion

With the present study, we evaluated the knowledge, beliefs, barriers and behaviors of MHP regarding exercise for people with mental illnesses combined with patients' perspective and sex differences in patients' exercise participation using the EMIQ-G.

### 4.1 Knowledge regarding the benefits of exercise (MHP and patients)

The results of the “knowledge” section indicate that both groups (MHP and patients) already knew a lot about the benefits of exercise. Contrary to other studies (22, 36, 37), we addressed the physical benefits of regular exercise including cholesterol level, blood pressure, depression and risk of cancer. Our data showed that MHP knew more than patients. This finding suggests a relationship between education level and knowledge about physical activity since our MHP had higher levels of education than the patients. Nevertheless, gaps in knowledge about the very basic effects of physical activity on several disorders can be recognized, e.g., only 67% knew that exercise can prevent the development of chronic diseases, only 85% knew that physical activity can lower total blood cholesterol levels, only 79% of MHP are aware of physical activity in the prevention of depression and only 60% knew for sure that physical activity may reduce the risk of cancers.

However, most MHP (78.0%) reported that they had never received any formal training in exercise prescription, which is in line with a previously published study that examined MHP's knowledge, attitudes and behaviors regarding exercise for patients with a mental illness in forensic psychiatric inpatient care (22). The authors revealed that out of the 239 participants, only 39 (16.3%) stated that they had undergone formal training in exercise prescription emphasizing that expertise in exercise is of importance to patient adherence (22). Of our 19 MHP (19.6%) that had received formal training, all referred to their professional education when asked to specify the formal training. However, most of our employees were still untrained which is also criticized in another study that evaluated 73 MHP's knowledge about exercise in people with mental illness in Brazil (23). The authors suggested that it is necessary to include physical activity and exercise training in mental health curricula.

Due to the importance of exercise in mental illness treatment, all MHP should acquire sufficient knowledge to be able to recommend a more active lifestyle for their patient. Handing out brochures about the positive effects or even concrete training recommendations could be a useful tool for promoting more exercise as it is time efficient, easy to distribute and realizable in a short period of time.

### 4.2 Mental health professionals' perspective

#### 4.2.1 Beliefs regarding exercise

Medication was ranked as the most important therapeutic option by MHP, while exercise was ranked scarcest on first position. This is in line with another study which stated that exercise is important, but also named medication first (23). Nevertheless, with increasing recognition of the benefits of exercise, we emphasize the importance of integrating exercise into clinical practice guidelines as an essential complement to the treatment of mental illnesses (38).

#### 4.2.2 Behavior of exercise prescription

Despite only part of the MHP (19.0%) stated that they had undergone formal training, they prescribed exercise *most of the time* (48.4%) or *always* (37.9%). This is especially positive comparing data in severe mental illnesses (23) and forensic psychiatric care (22) where partially 40%–50% of MHP never prescribe physical activity. However, it seems that brochures were hardly used and especially the MHP group of nurses used “referral to an exercise professional” more often than other methods of promoting exercise. Developing specific booklets might be a low-threshold method in order to provide helpful information and recommendation for exercise in people with mental illnesses. Further, our MHP seemed to give different recommendations for exercise regarding the type, duration, and intensity of the activity, which apparently do not follow current guidelines. It would be more consistent to provide standardized recommendations (e.g., WHO guidelines), with the option of individual adjustment for psychiatric patient. Following the FITT (frequency, intensity, time, type) principle (39) may facilitate MHP to recommend consistent training instructions, as previously indicated by Lee et al. (40).

#### 4.2.3 Barriers to exercise prescription

In general, the barriers inquired were not very high. More than 85.0% of the MHP *Strongly Disagree* (52.1%) or *Disagree* (34.0%) with the statement that prescribing exercise to people with a mental illness is not part of their job. This is consistent with previous research (37, 41, 42), showing nurses working in mental health would believe that exercise prescription is part of their role. However, in contrast to other studies (43, 44), respondents in the present study indicated that current workloads are not a barrier to exercise prescription (50.0% *Strongly Disagree* and 40.4% *Disagree*). This is an important finding since previous studies suggested that exercise prescription would extend to systemic barriers such as competing priorities, limited resources and fragmentation of the health care system in which they work (18, 43, 45). Despite the reported low organizational barriers in prescribing exercise in our study, the potential of exercise within the multidisciplinary treatment needs to be further developed. Brochures and the training of individuals (e.g., one expert on every ward) could help to overcome possible barriers.

### 4.3 Patients' perspective

#### 4.3.1 Motivating factors and barriers towards exercise

When being regularly active in health sports, our patients expected consistently positive effects. In accordance with previous finding (46, 47), our patients tended to be less active and had a higher BMI than the general population/MHP. Quantitative studies providing data on motivating factors towards exercise among patient with mental illnesses established *losing weight* (83.0% of patients), *improving mood* (81.0%) and *reducing stress* (78.0%) as the most common motivation (15). However, according to our data, there might be a higher level of interest in being more active with a financial reward (e.g., bonus in health insurance, free or discounted gym membership), with detailed training counselling and regular exercise support. Supervision by trained exercise professionals is a factor for stable adherence rates in people with depression (48). Patients with mental illnesses experienced several barriers that would make it difficult to take full advantage of the benefits associated with physical activity. The results revealed that patients agreed with several statements located at socio-ecological level where stigma and lack of support from their family and friends were considered as major barriers to engage in exercise. Social support is a potential moderator catching patients' volition to exercise. It has been shown that those who exercise with others may have extra health benefits compared to exercising alone (49). Thus, it might be of great benefit encouraging patients to exercise with friends or family (50) or providing group activities in a mental health setting.

#### 4.3.2 Sex differences

Concerning sex differences in patients, our data revealed interesting evidence. Males seemed to experience exercise more often as a positive distraction than females and seemed to have less physical health barriers to exercise (e.g., feeling unwell, feeling too fat). In some patients, depressive episodes present with higher values of irritability and anger rather than sadness and low self-esteem, which is more often the case in male patients (51). Our data have two main implications in this regard: first, in order to find ways to manage impulsiveness and inner restlessness, exercise programs with high intensity might be helpful in reducing such symptoms. This could be the reason that male patients in our cohort experienced more positive distractions from exercise. Second, developing exercise activities in order to overcome low self-esteem and physical health related barriers (more often named by female participants), activities done with others of the same gender (e.g., only-women groups) or at the same level of ability, exercising alone or with low intensity might be helpful for this female patient group (52). In summary, our findings could help to personalize our training recommendations in order to target more specific symptoms and thereby to motivate more patients to exercise.

### 4.4 Limitations

Some limitations need to be considered when interpreting our data. First, all results must be interpreted with caution because more female MHP and patients participated in the study. Some of our results (EMIQ, demographics) are based on self-reported data, derived from questionnaires and surveys administered to patients and MHP. Therefore, the results could be affected by response bias, or participations lacking interest or experience with exercise to accurately describe knowledge, barriers or motivation factors. We measured daily activity level in patients with mental illness only by the questionnaire IPAQ-SF (32). Although there are high correlations between this questionnaire and accelerometers (33), the assessment of physical activity was self-reported in this study and patients may have been inclined to overrate, for example, moderate activities as vigorous activities, as previously discussed by Petzold et al. (53). For future studies and individual diagnosis, the authors recommend a combination of both self-reported and objective measurements at the same time (53). We could not explore the differences between MHP in detail due to a small number of participants from each profession group. Future studies should investigate these limitations and include a greater number of MHP from all areas. It should also be considered that the large majority of patients (92.0%) had a diagnosis of affective disorders, while schizophrenia, dissociative disorders, eating disorder and mental and behavioral disorders were relatively under-represented among our included patients. Thus, future research should examine if the same identified barriers and motivation factors towards exercise also generalize to patients with other mental illnesses. Additionally, the positive results from MHP statements (e.g., high frequency of exercise prescription) can possibly be explained because clinical staff with high interest in exercise or physical activity were more likely to participate in this study.

## 5 Conclusion

Exercise offers great potential to improve mental and physical health of people with mental illnesses. There is now an abundance of data demonstrating that physical activity needs to be more integrated, recommended and prescribed in the clinical context of treating mental illnesses. However, due to this importance, all MHP should acquire sufficient knowledge to be able to educate the positive effects of exercise and to recommend a more active lifestyle for their patients. Additionally, being/becoming physically active and seeing oneself responsible as a role model may increase the authenticity of physical activity recommendations and facilitate exercise uptake.

In light of the results of the present study as well as based on research on the positive effects of exercise for patients with mental illness in general, in addition to the implementation of these findings within concrete recommendations in therapeutic guidelines, strategies can help to assist uptake and continuation of exercise in order to consider the benefits of exercise beyond the physical domain:

(1) Educating MHP and patients on the potential benefits of physical activity in a time efficient and easily accessible way, e.g., via psychoeducative brochures.
(2) Providing consistent exercise recommendations (e.g., WHO guidelines).
(3) Adapting exercise prescription should account for personal preferences and previous experiences in terms of making it the most enjoyable experience.
(4) Offering specific exercise intervention, e.g., supervised exercise interventions, training with detailed guidance and regular support or financial reward for physical activity.
(5) Developing exercise activities that address specific symptoms, e.g., high intensity workouts to manage impulsiveness and inner restlessness (more often seen in male patients with depression) or low-intensity groups/smaller groups/female-only groups to overcome low self-esteem and health-related barriers (more often seen in female patients with depression).
(6) Encouraging patients to exercise with family and friends or to participate in peer groups.

Increased use of exercise in psychiatric care, individually adapted to the patients' needs is important in order to address psychiatric symptoms.

## Data Availability

The datasets generated for this study are available on request to the corresponding author.
