# Supplementary material for: Implementing exercise recommendations into clinical practice—new findings from mental health professionals' and patients' perspectives in a university psychiatric setting
Source: Front Sports Act Living. 2024 May 20;6:1336356. doi: 10.3389/fspor.2024.1336356 (PMC11144853; doi:10.3389/fspor.2024.1336356)
Supplement: Supplementary file 1 [file Datasheet1.pdf]

Supplementary Material

S1

EMIQ-G

Fragenbogen zur körperlichen Aktivität bei psychischen Erkrankungen

Wissen, Einstellungen und Verhalten bezüglich körperlicher Aktivität bei Menschen mit psychischen Erkrankungen

Version für Patienten

Dieser Fragebogen stellt Fragen zu Ihrem Wissen, zu Ihren Einstellungen und zu Ihren Verhaltensweisen bezüglich körperlicher Aktivität bei Menschen mit einer psychischen Erkrankung. Wir bitten Sie, alle Fragen vollständig zu beantworten. Es gibt keine richtige oder falsche Antwort und es ist wichtig, dass wir eine Antwort erhalten, die Ihre Sicht als **Patient** widerspiegelt. Im Rahmen dieses Fragebogens umfasst der Begriff ‚psychische Erkrankung' alle psychischen Erkrankungen, einschließlich aber nicht beschränkt auf Depression, Schizophrenie, bipolare Störung I und II, posttraumatische Belastungsstörung und andere psychische Erkrankungen.

Teil 1. Wissen.
Dieser Abschnitt stellt Fragen über Ihr Wissen bezüglich der Vorteile von körperlicher Aktivität

[Due to copyright not attached.]

Teil 2. Barrieren, die Menschen mit einer psychischen Erkrankung bei der Teilnahme an körperlichen Aktivitäten im Weg stehen können

Inwiefern stimmen **Sie** den folgenden Aussagen zu, die Bezug nehmen auf Barrieren bei körperlicher Aktivität für Menschen mit psychischen Erkrankungen?

7. Meine psychische Gesundheit macht es mir unmöglich, körperlich aktiv zu sein.

1	2	3	4	5
Stimme überhaupt nicht zu	Stimme nicht zu	Weder Zustimmung noch Ablehnung	Stimme zu	Stimme voll und ganz zu

8. Ich bin besorgt, dass körperliche Aktivität meinen Zustand verschlechtern könnte.

1	2	3	4	5
Stimme überhaupt nicht zu	Stimme nicht zu	Weder Zustimmung noch Ablehnung	Stimme zu	Stimme voll und ganz zu

9. Ich glaube nicht, dass körperliche Aktivität mir bezüglich meiner psychischen Erkrankung helfen wird.

1	2	3	4	5
Stimme überhaupt nicht zu	Stimme nicht zu	Weder Zustimmung noch Ablehnung	Stimme zu	Stimme voll und ganz zu

10. Meine körperliche Gesundheit macht es mir unmöglich, körperlich aktiv zu sein.

1	2	3	4	5
Stimme überhaupt nicht zu	Stimme nicht zu	Weder Zustimmung noch Ablehnung	Stimme zu	Stimme voll und ganz zu

11. Ich bin besorgt, dass ich mich während der körperlichen Aktivität verletzen könnte.

1	2	3	4	5
Stimme überhaupt nicht zu	Stimme nicht zu	Weder Zustimmung noch Ablehnung	Stimme zu	Stimme voll und ganz zu

12. Es geht mir zu schlecht, um mich körperlich zu betätigen.

1	2	3	4	5
Stimme überhaupt nicht zu	Stimme nicht zu	Weder Zustimmung noch Ablehnung	Stimme zu	Stimme voll und ganz zu

13. Es nimmt zu viel Zeit in Anspruch.

1	2	3	4	5
Stimme überhaupt nicht zu	Stimme nicht zu	Weder Zustimmung noch Ablehnung	Stimme zu	Stimme voll und ganz zu

14. Psychisch krank zu sein, ist mit zu viel Stigmatisierung behaftet.

1	2	3	4	5
Stimme überhaupt nicht zu	Stimme nicht zu	Weder Zustimmung noch Ablehnung	Stimme zu	Stimme voll und ganz zu

15. Ich weiß nicht, was ich tun soll.

1	2	3	4	5
Stimme überhaupt nicht zu	Stimme nicht zu	Weder Zustimmung noch Ablehnung	Stimme zu	Stimme voll und ganz zu

16. Meine Freunde oder Familie sind nicht gemeinsam mit mir körperlich aktiv.

1	2	3	4	5
Stimme überhaupt nicht zu	Stimme nicht zu	Weder Zustimmung noch Ablehnung	Stimme zu	Stimme voll und ganz zu

17. Es gibt zu viele Nebenwirkungen von Medikamenten.

1	2	3	4	5
Stimme überhaupt nicht zu	Stimme nicht zu	Weder Zustimmung noch Ablehnung	Stimme zu	Stimme voll und ganz zu

18. Mir fehlt das Selbstvertrauen, um körperlich aktiv zu sein.

1	2	3	4	5
Stimme überhaupt nicht zu	Stimme nicht zu	Weder Zustimmung noch Ablehnung	Stimme zu	Stimme voll und ganz zu

19. Ich bin zu dick, um körperlich aktiv zu sein.

1	2	3	4	5
Stimme überhaupt nicht zu	Stimme nicht zu	Weder Zustimmung noch Ablehnung	Stimme zu	Stimme voll und ganz zu

20. Ich habe zu viele Probleme mit meiner körperlichen Gesundheit.

1	2	3	4	5
Stimme überhaupt nicht zu	Stimme nicht zu	Weder Zustimmung noch Ablehnung	Stimme zu	Stimme voll und ganz zu

21. Es gibt keinen sicheren Ort für mich, an dem ich körperlich aktiv sein kann.

1	2	3	4	5
Stimme überhaupt nicht zu	Stimme nicht zu	Weder Zustimmung noch Ablehnung	Stimme zu	Stimme voll und ganz zu

22. Ich habe keine Ausrüstung, mit der ich körperlich aktiv sein kann.

1	2	3	4	5
Stimme überhaupt nicht zu	Stimme nicht zu	Weder Zustimmung noch Ablehnung	Stimme zu	Stimme voll und ganz zu

Teil 3. Menschen mit psychischen Erkrankungen berichten aber auch von Anreizen, die unterstützend bei der Ausübung körperlicher Aktivität wirken. Inwieweit stimmen Sie diesen Aussagen zu?

23. Eine finanzielle Belohnung für körperliche Aktivität (z. B. über Bonusprogramm der Krankenkasse) würde ich als Anreiz empfinden?

1	2	3	4	5
Stimme überhaupt nicht zu	Stimme nicht zu	Weder Zustimmung noch Ablehnung	Stimme zu	Stimme voll und ganz zu

24. Eine ausführliche Beratung zur Ausführung der körperlichen Aktivität würde mir die Ausübung erleichtern.

1	2	3	4	5
Stimme überhaupt nicht zu	Stimme nicht zu	Weder Zustimmung noch Ablehnung	Stimme zu	Stimme voll und ganz zu

25. Wenn ich bei der Ausübung der körperlichen Aktivität regelmäßig unterstützt und begleitet werde, fällt es mir leichter, die körperliche Aktivität in meinen Alltag zu integrieren.

1	2	3	4	5
Stimme überhaupt nicht zu	Stimme nicht zu	Weder Zustimmung noch Ablehnung	Stimme zu	Stimme voll und ganz zu

Teil 4. Der folgende Abschnitt enthält Fragen zu Ihren Erwartungen an die Konsequenzen, wenn Sie eine körperliche Aktivität ausüben. Inwieweit stimmen Sie diesen Aussagen zu?

26. Wenn ich regelmäßig gesundheitssportlich aktiv bin, reduziert sich mein Medikamentenbedarf.

1	2	3	4	5
Stimme überhaupt nicht zu	Stimme nicht zu	Weder Zustimmung noch Ablehnung	Stimme zu	Stimme voll und ganz zu

27. Wenn ich regelmäßig gesundheitssportlich aktiv bin, verbessern sich meine gesundheitlichen Werte.

1	2	3	4	5
Stimme überhaupt nicht zu	Stimme nicht zu	Weder Zustimmung noch Ablehnung	Stimme zu	Stimme voll und ganz zu

28. Wenn ich regelmäßig gesundheitssportlich aktiv bin, lerne ich nette Leute kennen.

1	2	3	4	5
Stimme überhaupt nicht zu	Stimme nicht zu	Weder Zustimmung noch Ablehnung	Stimme zu	Stimme voll und ganz zu

29. Wenn ich regelmäßig gesundheitssportlich aktiv bin, verbessert sich mein körperliches Wohlbefinden.

1	2	3	4	5
Stimme überhaupt nicht zu	Stimme nicht zu	Weder Zustimmung noch Ablehnung	Stimme zu	Stimme voll und ganz zu

30. Wenn ich regelmäßig gesundheitssportlich aktiv bin, verbessert sich mein Selbstbewusstsein.

1	2	3	4	5
Stimme überhaupt nicht zu	Stimme nicht zu	Weder Zustimmung noch Ablehnung	Stimme zu	Stimme voll und ganz zu

31. Wenn ich regelmäßig gesundheitssportlich aktiv bin, kann ich stolz auf mich sein.

1	2	3	4	5
Stimme überhaupt nicht zu	Stimme nicht zu	Weder Zustimmung noch Ablehnung	Stimme zu	Stimme voll und ganz zu

32. Wenn ich regelmäßig gesundheitssportlich aktiv bin, verringert sich mein Gewicht.

1	2	3	4	5
Stimme überhaupt nicht zu	Stimme nicht zu	Weder Zustimmung noch Ablehnung	Stimme zu	Stimme voll und ganz zu

33. Die körperliche Aktivität bedeutet für mich eine positive Ablenkung.

1	2	3	4	5
Stimme überhaupt nicht zu	Stimme nicht zu	Weder Zustimmung noch Ablehnung	Stimme zu	Stimme voll und ganz zu

34. Durch regelmäßige körperliche Aktivität wird mein Tagesablauf besser strukturiert.

1	2	3	4	5
Stimme überhaupt nicht zu	Stimme nicht zu	Weder Zustimmung noch Ablehnung	Stimme zu	Stimme voll und ganz zu

35. Wenn ich regelmäßig gesundheitssportlich aktiv bin, kann ich mich blamieren.

1	2	3	4	5
Stimme überhaupt nicht zu	Stimme nicht zu	Weder Zustimmung noch Ablehnung	Stimme zu	Stimme voll und ganz zu

36. Wenn ich regelmäßig gesundheitssportlich aktiv bin, kann ich mich überanstrengen.

1	2	3	4	5
Stimme überhaupt nicht zu	Stimme nicht zu	Weder Zustimmung noch Ablehnung	Stimme zu	Stimme voll und ganz zu

Wir danken Ihnen für Ihre Zeit. Falls Sie irgendwelche anderen Kommentare hinsichtlich der Empfehlung von körperlicher Aktivität für Menschen mit einer psychischen Erkrankung ergänzen möchten, können Sie dies an dieser Stelle gerne tun.

____

____
